# Post-Anthesis Water-stressed Barley Maintains Grain Specific Weight Through Altered Grain Composition and Plant Architecture

**DOI:** 10.3390/plants9111564

**Published:** 2020-11-13

**Authors:** Aaron Hoyle, Maree Brennan, Logan Rees, Gail E. Jackson, Stephen P. Hoad

**Affiliations:** 1 Scotland’s Rural College (SRUC), Edinburgh Campus, King’s Buildings, West Mains Road, Edinburgh, Scotland EH9 3JG, UK; Aaron.Hoyle@sruc.ac.uk (A.H.); mareebrennan@gmx.com (M.B.); 2 The University of Edinburgh, Crew Building, King’s Buildings, Alexander Crum Brown Road, Edinburgh, Scotland EH9 3FE, UK; Logan.Rees@sruc.ac.uk (L.R.); G.Jackson@ed.ac.uk (G.E.J.)

**Keywords:** barley (*Hordeum vulgare* L.), grain size, specific weight, water stress

## Abstract

Specific weight (SW) is a long-established measure used as a malting quality specification in barley, with an increased SW thought to result in a higher malt output. Specific weight is a product of individual grain density as determined by grain composition and structure, and grain packing efficiency in a container as determined by grain dimensions. We investigated the effect of moderate but prolonged post-anthesis water stress on barley plant and grain development using pots of cultivars with a known range of SWs to explore how altering plant growth influence SW. Water stress was expected to influence these grain characteristics through decreased photosynthetic capacity. We demonstrated that SW was maintained under water stress conditions through compensatory mechanisms such as increased tiller mortality which preserved grain physical parameters on the main shoots. However, water stress significantly affected plant development by reducing not only ear number and yield, but also grain filling duration, plant biomass and ear length. Grain composition was also altered, with water-stressed plants having reduced carbon:nitrogen. Therefore, although SW can be conserved under water-stressed conditions, grain composition and plant development are altered, producing smaller harvests with higher grain nitrogen content. This would result in bulks of malting barley having different malt outputs despite having the same SW.

## 1. Introduction

Barley (*Hordeum vulgare* L.) is an important cereal crop worldwide. In 2017 a total of 147 million tonnes were harvested globally and in the UK 7 million tonnes were harvested from 1.2 million hectares [1]. The majority of this is grown for feed and malting. In the UK, it is largely spring barley that is grown for malting, and it is required to attain certain grain quality specifications and if these are achieved a premium is paid compared with barley destined for feed. One of these quality specifications is specific weight (SW), a measure of the weight of grain per unit volume, and is measured in kilograms per hectolitre (kg hL^−1^). Specific weight is an established measure used throughout the cereal sector when grading quality. A high SW is thought to be indicative of higher quality grain which is associated with a high starch content, enhanced malt quality and/or malting efficiency [2]. Therefore, minimum SWs are specified in a contract between farmers and end users. Consequently, farmers often strive to select cultivars with an inherently high SW and employ agronomic techniques such as cultivar selection and fertiliser regimes to keep SW high.

Specific weight is a complex quality trait determined by two components: grain density (GD) and packing efficiency (PE) of the grain [3]. There is limited information concerning the grain parameters which determine these components. It is hypothesised that grain composition and internal structure influence GD, and grain size and morphology determine PE. Grain size is homogenized to some extent before malting, by passing grain through sieves with known slot sizes, to reduce inefficiencies caused by different sized grains malting at different rates. By weight, barley grains are composed of 60 to 80% starch, 9 to 13% protein, 10 to 15% water and 1 to 2% lipids [4]. It is these constituents that are thought to influence GD, and they are known to have impacts on the malting process and product yield. It is not only the absolute amount of these constituents that is thought to be important in malting but also the composition or fine structures of these molecules. For example, both the composition of starch in terms of the proportion of the polymers amylose and amylopectin, and ratios of A and B starch granules, impact the fermentable sugars produced in the malting process [5].

Previous work has shown that within a cultivar if grains are graded by GD, there is no relationship between starch concentration and GD [6]. Despite starch concentration not correlating with GD, there is evidence that starch composition may influence GD with the proportion of starch B granules positively correlating with GD [6]. Furthermore, higher density grains have an increased nitrogen (N) concentration, and this has been shown to explain nearly 50% of the observed variation in GD. An excessively high N concentration is undesirable for many malting uses [6]. This highlights the potential role of N concentration in increasing GD and consequently SW. Furthermore longer, deeper grains have a reduced PE, resulting in a reduced SW for these larger grains [3].

The yield of the barley crop is the weight of grains per area of crop harvested, and this is strongly correlated with grain number rather than grain size [7]. Spring barley, often used for malting, has only two rows of grains per ear, whereas winter barley has six rows of grains per ear, and the yields of spring barley are, therefore, typically lower than winter barley. Row-type is genetically determined, but crop management and environment may affect the number of ears and tillers (secondary shoots) which influence barley yield [7]. Other environmental conditions influence grain characteristics themselves, as the following examples show. High night time temperature after anthesis reduced grain weight and the duration of grain filling in barley [8]. Shading of barley was found to reduce the rate of grain filling, but not affect the duration of grain filling [9]. Grain weight and yield was reduced by a combination of post-anthesis water deficit and heat stress [10]. Post-anthesis water stress has been shown to decrease grain width, but not grain length, indicating that a reduction in grain filling affects grain width more than length [11]. Prolonged water stress throughout the grain filling period reduces the length of grain filling and yield [12]. Increased tiller mortality has been shown to be a significant contributor to the reduction in yield in water-stressed plants through reducing total grain number [12]. These stresses are likely to influence grain development and consequently the PE of the grain.

Alongside physical characteristics, chemical and biochemical processes are also impacted by water stress. Grain starch concentration has been shown to be lower under water-stressed conditions, negatively influencing yield [13]. Water deficit has also been shown to influence starch accumulation, composition, ultrastructure and functionality [14]. Barley starch composition is thought to be more resilient to water stress than other cereals, with changes only noticeable after a more severe stress [14]. Therefore, water stress is likely to change grain composition and, consequently, GD. In general water deficit throughout cereal development tends to result in a higher proportion of A-type starch granules; however, the timing of the stress alters its effects, making predictions of the stress effect on malting quality difficult [15].

In order to enhance our current understanding of how changing plant growth and grain parameters influence SW, a stress known to have relevant physiological consequences to grain composition, water stress, was used as a tool to impose changes in plant development. The effect of water stress on SW has not been studied before, and the understanding the mechanisms through which SW is achieved under changing environmental conditions is especially relevant due to climate change. Furthermore, quantifying the outcome of water stress on both components of SW and cultivars with varying SWs is necessary to understand how changes in SW relate to grain quality for different markets. The aims of this study were to investigate the effect of moderate but prolonged water stress during grain filling on the grain quality measure SW, and the mechanisms influencing this measure. This was achieved by the following objectives: (i) to establish how water stress modifies plant development, yield components and grain composition which impact on SW, (ii) to evaluate changes in SW according to its components and traits which affect them and (iii) investigate associations between grain parameters and the components of SW.

## 2. Results

### 2.1. Quantification of Water Stress and Plant Development

**Figure 1:**
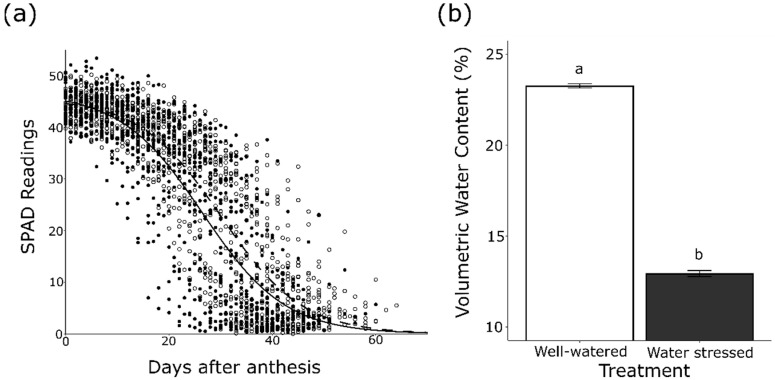
Effects of water stress imposed during plant growth. (**a**) The relationship between SPAD values and days after anthesis for experimental plants grown under well-watered (open circles) and water-stressed (filled circles) conditions. The dashed sigmoid curve represents pots grown under well-watered conditions and the solid sigmoid curve represents pots grown under water-stressed conditions. (**b**) The mean volumetric water content (*v*/*v*) of pots over the grain-filling period under well-watered and water-stressed treatments. Treatments that do not share a letter are significantly different. Error bars are standard error of the mean.

SPAD readings demonstrated that the water stress treatment resulted in a quickening in leaf senescence, indicating the water stress treatment successfully reduced the total duration of photosynthesis in these plants (Figure 1a). The treatment imposed significantly reduced water content from 23.27% in well-watered pots to 12.94% in water-stressed pots (*p* < 0.001, Figure 1b).

**Figure 2:**
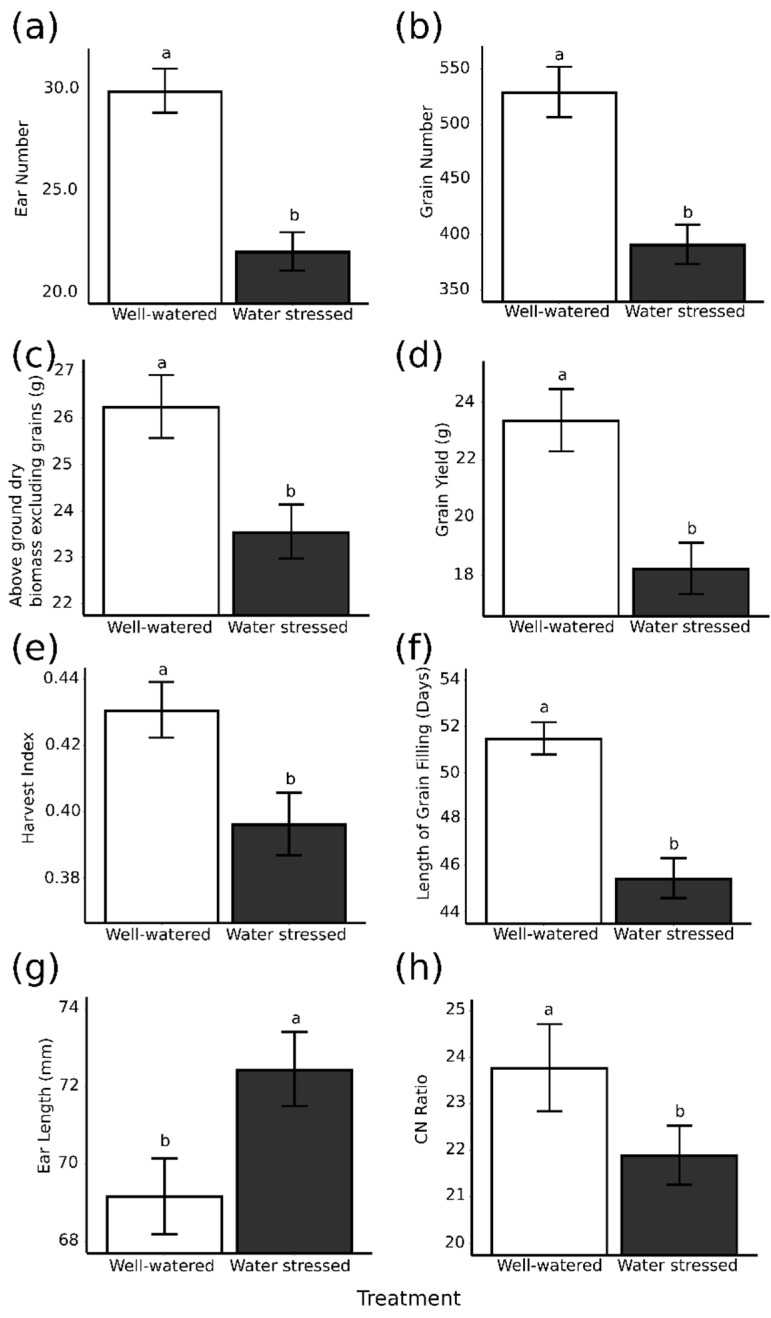
The significant effects of the well-watered (open bars) compared with water-stressed treatment (filled bars) on plant growth parameters: (**a**) ear number, (**b**) grain number, (**c**) above ground biomass excluding grains, (**d**) grain yield, (**e**) harvest index, (**f**) length of grain filling, (**g**) ear length and grain parameter (**h**) C:N ratio. Significant differences are indicated by different letters above bars (*p* < 0.05). Error bars are the standard error of the means.

Plant growth parameters were measured on the three cultivars Octavia, Concerto and Sienna under well-watered and water-stressed conditions. Mean values across the three reps for plant growth parameters measured are provided in Supplementary Table S1 with levels of significance in Supplementary Table S2. The water stress treatment decreased: ear number from 30 to 22 (*p* < 0.001, Figure 2a), grain number from 529 to 391(*p* < 0.001, Figure 2b), plant biomass from 26.25 g to 23.56 g (*p* < 0.001, Figure 2c), grain yield from 23.36 g to 18.26 g (*p* < 0.001, Figure 2d), harvest index from 0.43 to 0.40 (*p* < 0.01, Figure 2e) and the length of grain fill from 51 days to 45 days (*p* < 0.001, Figure 2f). Whereas the water stress increased ear length from 69.17 mm to 72.45 mm (*p* < 0.01 Figure 2g), there was no evidence that water stress had an effect on grain weight.

### 2.2. The Effect of Water Stress on Grain Parameters

Grain morphology, size and composition are key determinants of grain quality in malting barley. Detailed grain parameters which contribute to overall barley grain quality including grain size fraction distribution, screenings, dimensions, 2-D area, circularity, starch concentration, amylose/amylopectin, carbon and nitrogen concentrations were measured on the three cultivars Octavia, Concerto and Sienna under well-watered and water-stressed conditions. Mean values across the three reps for these grain parameters are provided in Table 1 with levels of significance in Supplementary Table S3.

Figure 2 shows that water stress had a significant effect on the ratio of carbon to nitrogen, as shown in Figure 2h. However, a post-hoc multiple comparison test could not distinguish between N concentration in well-watered (1.70%) and water-stressed (1.83%) grain. Well-watered plants did however have a significantly higher (*p* < 0.05, Figure 2h) carbon to nitrogen ratio with a ratio of 23.78 to a ratio of 21.90 for water-stressed grains. Starch and protein content per grain were also calculated; protein content varied significantly with treatment, but starch content did not. Well-watered pots had a significantly lower protein content per grain (*p* < 0.05) of 4.97 mg/grain compared to 5.45 mg/grain in water-stressed pots.

Grains which are large, uniform and bold are preferred for malting due to their beneficial characteristics, such as high starch concentration and high germination rate. Water stress did not have a significant effect on many grain parameters including size classes, despite drastically affecting overall grain yield. Grain length, grain width and grain depth also remained unchanged as a result of the water stress.

Specific weight and its components GD and PE were also measured. Neither SW nor its components were significantly affected as a result of the water stress, this is not surprising with the lack of differences observed in the other grain parameters which contribute to this measure.

### 2.3. The Effect of Cultivar on Grain Parameters

**Figure 3:**
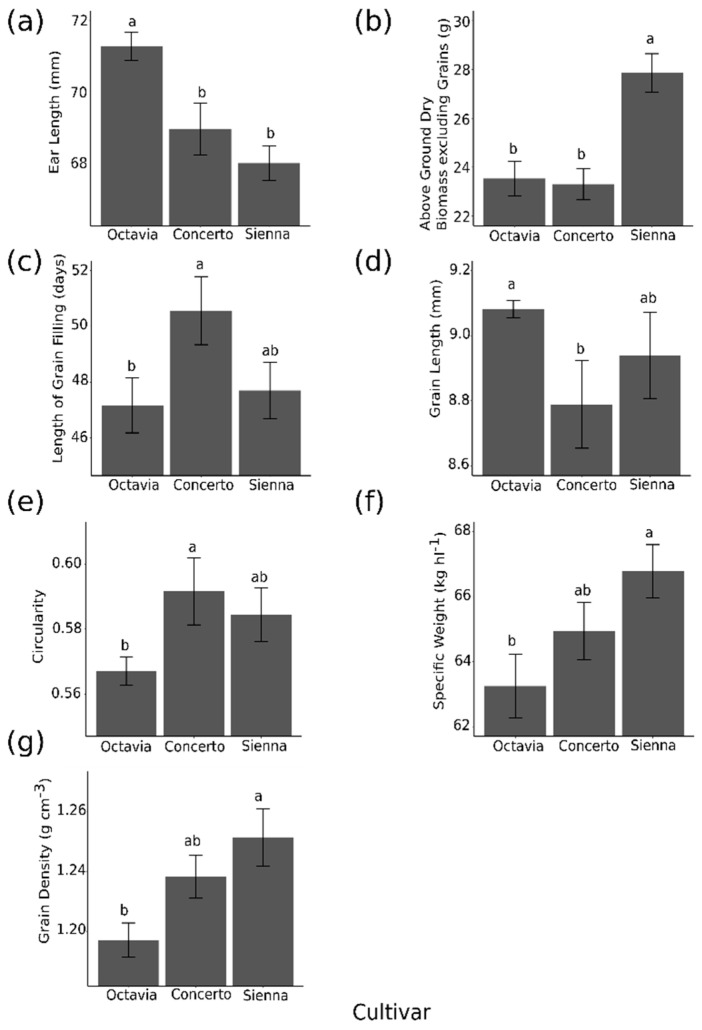
The significant effects of cultivar on plant growth parameters (**a**) ear length, (**b**) above ground dry biomass excluding grains, (**c**) length of grain filling and grain parameters: (**d**) grain length, (**e**) circularity, (**f**) SW and (**g**) GD. Significant differences are indicated by different letters above bars (*p* < 0.05). Bars are the standard error of the means.

As a result of different cultivars there was no significant difference in the grain size distribution. The significant effects of cultivar on grain parameters on a per pot basis are summarised in Figure 3. Significant differences were observed among the cultivars for ear length (Figure 3a), above-ground non-grain biomass (Figure 3b) and length of grain filling (time to ripen, Figure 3c). In terms of dimensions and morphology, cultivar had a significant effect on grain length with Octavia at 9.08 mm being significantly longer than Concerto at 8.79 mm (*p* < 0.05, Figure 3d), but Sienna’s grain length of 8.94 mm was not different to Octavia or Concerto. No differences were observed in other morphological measures such as width, depth and 2-D area across different cultivars. Cultivar did, however, have a significant effect on grain circularity with Concerto having the highest circularity 0.59 which is significantly higher than Octavia 0.57 (*p* < 0.01, Figure 3e). Sienna has a circularity of 0.58, but this did not differ significantly from the other two cultivars. In addition to these unchanged morphological characters of the grain, grain weight also remained unchanged as a result of water stress and cultivar. With very few morphological and size differences between the samples created in this study this aspect of grain quality was essentially unchanged.

Specific weight was significantly affected by the cultivar, Sienna having the highest SW with 66.78 kg hL^−1^ which is significantly greater than Octavia with 63.25 kg hL^−1^ (*p* < 0.001, Figure 3f). The SW of Concerto, 64.94 kg hL^−1^ was not significantly different to the other two cultivars. Specific weight was not significantly affected by the water stress. Packing efficiency, one component of SW, was not affected by cultivar, but GD (the other component of SW) was. Grain density was highest in Sienna with 1.26 g cm^−3^, which was significantly higher than Octavia with 1.19 g cm^−3^ (*p* < 0.01, Figure 3g). The GD of Concerto was 1.24 g cm^−3^ which was not significantly different from the other two cultivars. Cultivar had no effect on size fraction distributions.

### 2.4. Correlations with Components of Specific Weight

The significance of correlations between grain parameters were analysed and a matrix of the Pearson correlation coefficients (r) is provided in Table 2 and the corresponding *p*-values are in Supplementary Table S4. Grain weight was shown to be strongly positively correlated with width (r = 0.89, *p* < 0.01) and depth (r = 0.84, *p* < 0.01) but not length. Grain length was negatively correlated with grain dimensions width (r = −0.51, *p* < 0.05) and depth (r = −0.53, *p* < 0.05). Grain width and depth are highly positively correlated with each other (r = 0.95, *p* < 0.001). This demonstrates that grain width and depth are tightly correlated with each other, but grain length is not correlated with either. Grain 2-D area is positively correlated with length (r = 0.7, *p* < 0.01) and has a slightly weaker but still positive correlation with volume (r = 0.48, *p* < 0.05), but with neither width nor depth. Grain perimeter was positively correlated with length (r = 0.91, *p* < 0.01) and negatively so with depth (r = −0.53, *p* < 0.05). The strong positive correlation between circularity and grain weight (r = 0.64, *p* < 0.01) highlights that “plumper” grains weigh more. Longer grains tend to be less dense (r = −0.56, *p* < 0.05), this could be a result of the husk not filling entirely at the extremity of the grain.

**Figure 4:**
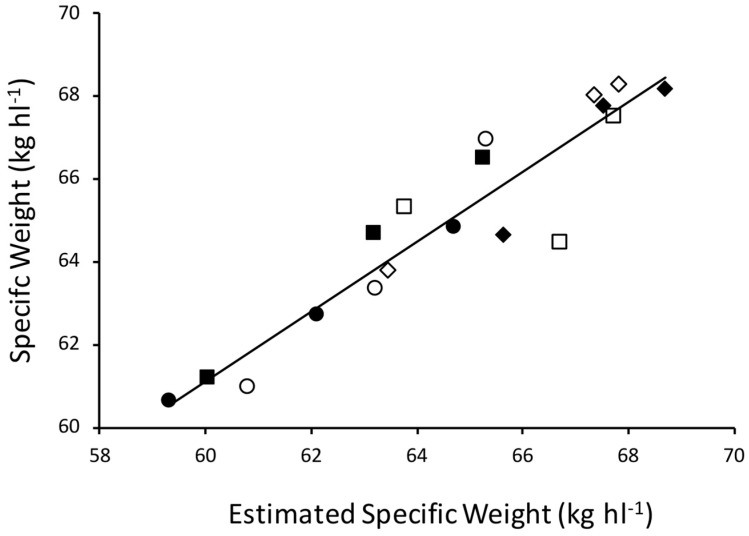
Specific weight of 18 barley grain samples plotted against the product of packing efficiency and grain density. The linear relationship is shown by the solid black line which has the equation, y = 0.84x + 10.15 (r = 0.94, *p* < 0.001). Solid shapes are well-watered, and open shapes are from water-stressed plants. Squares = Concerto, diamonds = Sienna and circles = Octavia.

A highly significant relationship between SW and the product of its components (r = 0.94, *p* < 0.001, Figure 4) PE and GD was observed. Grain PE positively correlated with grain weight (r = 0.62, *p* < 0.01) and depth (r = 0.62, *p* < 0.01). Specific weight correlates with all grain dimensions; positively so with width (r = 0.76, *p* < 0.001) and depth (r = 0.76, *p* < 0.001) and negatively so with length (r = −0.65, *p* < 0.01). Specific weight also positively correlates with grain volume (r = 0.53, *p* < 0.05) and circularity (r = 0.76, *p* < 0.001).

Nitrogen concentration is negatively correlated with grain width (r = −0.55, *p* < 0.05), grain depth (r = −0.49, *p* < 0.05) and circularity (r = −0.53, *p* < 0.05) highlighting that thinner more needle-like grains have higher nitrogen concentrations. The concentration of carbon has a strong positive correlation with nitrogen concentration (r = 0.68, *p* < 0.01). The concentration of starch was positively correlated with numerous grain parameters including: grain weight (r = 0.68, *p* < 0.01), width (r = 0.62, *p* < 0.01), depth (r = 0.59, *p* < 0.01), volume (r = 0.63, *p* < 0.01), PE (r = 0.48, *p* < 0.05) and SW (r = 0.76, *p* < 0.01). The only other variable that starch concentration correlates with is nitrogen concentration, with which it has a strong negative relationship (r = −0.76, *p* < 0.001). Amylose and amylopectin had no significant correlations with any other variables.

## 3. Discussion

Specific weight was maintained under water-stressed conditions; however, many aspects of plant development were altered by the water stress. A major effect of water stress on plant development was a reduction in ear number. Lower ear numbers had consequential effects on total grain number and yield, this is consistent with previous studies and is an important contributor to the reduction in yield experienced by water-stressed plants [12,16]. The ears that were left on water-stressed plants were longer, implying that tillers rather than primary shoots had an increased mortality. As such, it is likely that the water stress increased the duration of tiller mortality beyond flowering and into the beginning of grain development and filling. This has been observed previously in barley, where water stress applied at the beginning of grain filling caused not only a reduction in the number of fertile spikes but also significantly reduced the number of tillers per plant [12]. Plants were watered equally until anthesis, so this reduction in ear number but increase in ear length demonstrates a post-anthesis compensatory mechanism where tillers are aborted under water-stressed conditions.

Such a mechanism could allow plants to maintain a higher proportion of grains with higher grain weight when reduced photoassimilates are available under stressed conditions as a result of compromised photosynthesis. This response would be beneficial for plant progeny with more carbohydrates being available to fewer plant embryos instead of spreading resources more thinly across many embryos. Water stress also resulted in a shortening of the grain filling duration by six days and a reduction in above ground biomass; both are expected effects of water stress on cereal development [12,17]. That the mean grain weight was found not to be affected by water stress in the present study despite the shortened length of grain filling was likely due to the larger number of secondary tillers in the non-stressed plants containing smaller grains and lowering the average grain weight, rather than an increase in grain filling rate of the water-stressed plants. Yield and above ground biomass did not decrease by the same proportions because water stress reduced harvest index, demonstrating a reduction in reproductive efficiency as a result of stress. Similarly, harvest index has been shown to be lowered in rice when subject to a shading treatment, although unlike water deficit the shade treatment lengthened the grain filling period highlighting the contrasting effects on development of different stresses [18]. A restricted water supply is known to cause osmotic stress in plant cells and consequently cell damage contributing to premature leaf senescence, whereas plants under shaded conditions receive less photosynthetically active radiation reducing plant growth and the rate of grain filling. In field conditions water stress is often accompanied by a heat stress, although a heat stress was not applied in this study others have demonstrated the extreme negative impact of this stress combination at grain filling on spring barley yields [19]. Additionally, the timing of this stress has been shown to affect both spring barley yield and malt quality. When spring barley plants are subjected to these stresses yield and quality are affected to a more significant degree than when stresses are implemented at heading stages, in comparison to the vegetative growth stages [20].

Alongside water stress, different cultivars also resulted in significant differences in plant development. Cultivars varied in ear length, biomass and the length of grain fill. Although studying plant development is important to understand the effects of water stress and cultivar, it is the way in which development changes grain parameters that is of most importance to enhance the understanding of SW. Grain morphology was influenced by cultivar but not treatment with Octavia having longer, less circular grains in comparison to Concerto. The existence of a cultivar effect on grain morphology is consistent across different cereal species, demonstrating the genetic basis of grain dimensions, which is controlled by multiple genes, known as quantitative trait loci (QTLs) [21]. The SWs of cultivars followed the same rank order as parent material, highlighting the strong and consistent genetic influence on SW. Quantitative trait loci have been detected which are associated with grain size in barley, and with one of the components of SW being PE which is highly influenced by grain dimensions, it is likely that there are QTLs in barley which partly control SW [22]. Therefore, with SW remaining a breeding target, research into discovering these related QTLs would provide a useful molecular genetic basis for improving this measure of grain quality. The same rank order was exhibited by GD for the three cultivars, this and how GD correlates more strongly with SW than PE suggests that GD may contribute more proportionally to SW. Previous work has shown that GD contributed to 48.5% of the variation in SW and PE to 36.5% further highlighting a slight dominance in GD over PE in determining SW [3].

Alongside plant development water stress also influenced grain composition with a reduction in the C:N, and an increase in N content and protein content per grain. Grain composition is thought to be impacted by environmental conditions to a greater extent than morphology; despite this, QTLs have been identified for grain protein concentration. With nitrogen concentration known to significantly affect GD it is likely these QTLs are also related to SW, which could contribute to molecular breeding. Furthermore this work has emphasised that grain morphology and composition are related so should be discussed in tandem [23]. Strong correlations between starch concentration and grain width, depth and volume indicate that either starch accumulation in grains can result in these plumper grains, or plumper grains facilitate an enhanced storage of photoassimilates. Numerous genes have been identified in rice which are associated with grain shape: GRAIN SIZE 3 is a QTL for rice grain length and weight, GRAIN WIDTH 2 and GRAIN WIDTH 8 are QTLs for width and weight [21].

Mean grain weight was not affected by the water stress treatment. Although this was an unexpected result, it can be explained by the ways in which stress altered plant ear number per plant and grains per ear (sink capacity) and a plant’s ability to accumulate photoassimilates (source capacity). Despite having reduced source capacity, the water-stressed plants maintained mean grain weight in a smaller sink capacity or a reduced total grain number. Previous studies on cereals have shown that despite a reduced grain filling period as a result of water stress, the rate of grain filling can also be enhanced through increased remobilization of stem carbohydrates [24]. In other studies when water stress was imposed pre-anthesis grain yield, number and weight were reduced and total sink can be limited [25,26]. These studies indicate timing is crucial in determining how stress to the plant impacts on grain quality. Interestingly, maintenance of green-leaf area or “stay-green” traits have been shown to buffer grain quality against the detrimental effects of water stress [27].

The multifaceted nature of how many grain parameters influence SW is apparent in this analysis with SW significantly correlating with 10 different grain parameters. The results reinforced previous studies which have shown how SW is determined by changes in PE and GD, with a very strong relationship between the product of these and SW which is maintained across well-watered and water-stressed conditions [3]. It is clear from these pot trials that plants have a range of mechanisms that allow them to maintain high grain viability in the face of stress, and it would be very interesting to determine if, and to what extent, these mechanisms are deployed under water-stressed conditions in the field. A new finding was that wider and deeper grains have a higher PE, indicating plumper grains can pack more efficiently, this has previously been associated with SW but not PE [28]. A negative correlation between GD and grain length needs further investigation though it could be a result of long grains with a greater storage capacity being less filled with starch. This would be consistent with the positive correlation between grain width, depth and starch concentration. Furthermore, long grains in which the caryopsis was not extended to the full distal end of the husk would negatively contribute to GD. The traditional opinion that SW is associated with an increased starch concentration in the grain is supported by this study.

## 4. Materials and Methods

### 4.1. Plant Material and Growth

The three spring malting barley cultivars (Octavia, Concerto and Sienna) used in this study were selected due to their different SWs. According to the Agriculture and Horticulture Development Board’s (AHDB’s) recommended list 2016; Octavia is a low SW cultivar, Concerto intermediate and Sienna high, with reported SWs of 66.7, 68.8 and 70.7 kg hL^−1^, respectively. Seed was sourced from the AHDB and had been grown in the 2016 Recommended List trial in Docking, Norfolk, UK, under natural rainfall conditions. Experimental plants were grown under glasshouse conditions at Scotland’s Rural College, Edinburgh, from November 2016 to June 2017, in triplicate experiments as described below. The three experiments were sown on 21st November 2016, 18th January 2017 and 1st March 2017, respectively. From November until the end of April, plants were grown in a heated glasshouse (min temperature 16 °C). When natural daylight hours were insufficient light was supplemented artificially using 400 W sodium lights to give 16 h days with a photosynthetically active radiation at plant ear level of 150 µmol^−2^ s^−1^ at the plant ear level. From 1st May plants were moved into an unheated glasshouse with no supplementary light. Seven grains of each cultivar were sown into each of 10 separate 5 L pots and grown in Levington’s Advance M3 High Nutrient Potting compost containing ratios of 204 N, 104 *p* and 339 K (Levington Horticulture, Ipswich, UK). Any seeds that had not germinated within five days were replaced to ensure a density of seven plants per pot. Pot density was approximately six pots per square metre; therefore, plant density was ~42 plants per square metre. For each cultivar five of these pots were randomly allocated to a water stress treatment and five to a well-watered control treatment for each cultivar. There were, therefore, 30 pots in total. The five pots per treatment were expected to yield sufficient grain for one measurement of SW using a scaled down method comparable to industry standards [3]. A complete randomized block design with five blocks was used, with each cultivar: treatment combination represented once in each block and randomly assigned a position. The experiment was conducted three times in order to obtain replication with pot order randomly re-assigned in each block for each repetition.

### 4.2. Water Stress Treatment

All plants were grown under the same non-stressed conditions and watered daily until half of the main shoots in a pot reached anthesis, defined as growth stage 61 [29]. At this point pots were watered to field capacity and allowed to drain overnight and after which differential treatments were applied. Following anthesis, soil moisture readings were taken at least 6 days a week using a SM150 dielectric soil moisture sensor (Delta-T Devices Ltd.) with an attached HH150 hand moisture meter. Soil moistures were recorded as electrical conductivity (mV) from an average of three readings per pot and the manufacturers calibration used to equate this to volumetric water content. If a daily reading for volumetric water content was below 21% for well-watered pot, or below 10% for a water-stressed pot, water was added to increase soil moisture above these threshold values. In addition, at least every three days chlorophyll readings were taken using a SPAD-502 chlorophyll meter (Minolta, Japan), which measures the difference between transmittance through the leaf of red (650 nm) and infrared (940 nm) light. Readings were taken a third of the way up the penultimate leaf away from the main stem, described as leaf 2 [7]. For each pot the average of three readings from three labelled leaves was recorded. The experiment proceeded in this way until maximum grain dry weight had been reached for at least half of the main shoots in a pot [30]. At this point all watering was stopped and plants were allowed to dry out prior to harvest.

### 4.3. Plant Growth Measurements

Plants were hand threshed and numerous measurements taken per pot. These were grain weight (±0.0001 g), ear number per pot, ear length (±1 mm), grain number per pot, grain number per ear, spike fertility, which is measured as a proportion of total florets to grains, days of grain fill, plant above ground biomass (±0.01 g) and grain yield (±0.0001 g). Grain moisture per pot was estimated by drying two centrally located grains in an ear from each pot in an oven at 130 °C for 20 h and calculating the percentage weight loss from wet to dry. To enable harvest index to be calculated, shoots were dried at 70 °C for 48 h and weighed to give dry shoot biomass. Harvest index was then calculated as the ratio of harvested dry grain to total above ground shoot dry biomass.

### 4.4. Grain Sampling

Grain from each treatment and cultivar combination in each of the five blocks was pooled to give grain samples large enough to make SW measurements. Grain samples were cleaned by screening over a slotted 2.25 mm sieve with 19.05 mm long slots. SW was measured on this pooled grain using a scaled down published method which corresponds to the industry standard method [3]. Briefly, a 25 mL measuring cylinder of known total volume (39.16 mL) was filled with grain, and levelled using a straight edge, allowing calculation of weight per volume and conversion to the standard SW unit kg hL^−1^. The packing efficiency was calculated as the proportion of space in this cylinder occupied by grain. The mean grain number from three-cylinder re-fills was calculated, followed by multiplying this value by mean grain volume (see below), giving the packing efficiency. For further analysis to be conducted on grain samples from each cultivar and treatment combination a representative sample of grain was obtained by sequentially sieving each sample into size fractions using a stack of slotted 3.25, 3.00, 2.75 and 2.50 mm sieves, with 19.05 mm long slots. The weight of grain retained by each sieve fraction: extra-large (>3.25 mm), large (3.25 to 3.00 mm), medium (3.00 to 2.75 mm), small (2.75 to 2.50 mm) and extra small (2.50 to 2.25 mm) was weighed (accuracy ± 0.01 g). Three 100-grain samples were weighed from each fraction to estimate the mean grain weight in each fraction. This was used to estimate the total number of grains in each fraction, and a number proportional to the total number of grains from each fraction were chosen at random to give two separate 100-grain samples with grain sizes representative of the original bulk.

### 4.5. Grain Morphometrics and Specific Weight Components

In the first 100-grain sample, each grain was weighed, and the grain dimensions length, width and depth were measured using a hand-held digital caliper (±0.01 mm). These grains were placed onto an Epson Expression 836XL flatbed scanner alongside a ruler for scale. The 2-D area of the grains was estimated through image analysis in ImageJ [31]. Circularity was calculated in ImageJ as 4π(area/perimeter2). A value of 1.0 represents a perfect circle, the closer to 0.0 the more the shape represents an elongated polygon. Grain volume was measured on this grain sample using Archimedes’ principle with the weight of water being displaced by a grain being equal to the volume of grain. Each grain was weighed (±0.0001 g) then submerged in a beaker of water on the balance using a 0.5 mm× 25 mm hypodermic needle to submerge the grain.

### 4.6. Compositional Analysis

The second 100-grain sample was milled into a fine powder using a ball mill (Mixer Mill MM 200, Retsch, Germany). A FLASH 2000 Organic Elemental Analyzer (Thermo Scientific) was used to determine the proportion of carbon and nitrogen in the grain, usually referred to as the carbon and nitrogen concentration. Nitrogen is converted to protein by multiplying by the factor 6.25. The concentration of starch and the ratio of amylose and amylopectin were measured using Megazyme kits as previously described [30,32].

### 4.7. Data Analysis

All data were analysed in the open-source R software using version 3.4.1 [33]. Linear mixed-effects model (LMM) analysis was used via the restricted maximum likelihood algorithm (REML) in the “lme4” package [34]. An LMM was fitted for each measured trait in the experiment, listed as plant/grain parameters in Table 1. Cultivar, water stress treatment and their interaction were fixed effects, and the replicate number of the experiment was the random effect. Model fits were compared by likelihood ratio tests using the analysis of variance (ANOVA) function on hierarchical models, and non-significant variables (α = 0.05) were dropped sequentially until a minimally adequate model was reached for each parameter. The “emmeans” package was then used to calculate 95% confidence intervals to determine significant differences among samples [35]. Only the explanatory variables required by the minimally adequate model were tested; therefore, letters of significance are the same within either treatment or cultivar in the cases where the interaction term was not significant, and only one of the individual explanatory variables was significant.

## 5. Conclusions

Barley SW can be maintained in response to water stress by compensatory response mechanisms. However, despite SW being maintained, this does not necessarily mean the grain from water-stressed plants is of the same quality. This was demonstrated by the decrease C:N and increased N concentration in grains from water-stressed plants, despite there being no difference in SW. The observed increase in N concentration from 1.70 to 1.83% under water-stressed conditions is appreciable in the malting industry. For example, according to the Maltsters Association of Great Britain this increase would result in this grain being rejected for the brewing industry with targets set of 1.60 to 1.75%. Therefore, this work has continued to highlight the complexity of SW and its use as a malting quality criterion.

## Figures and Tables

**Table 1:** Summary of mean values ± standard deviations for grain quality parameters across three reps for the three cultivars and two treatment levels used in this study ^1^.

Grain Quality Parameters	Octavia		Concerto		Sienna	
	Well-Watered	Water Stress	Well-Watered	Water Stress	Well-Watered	Water Stress
**Size Classes**						
>3.25 mm	8.29 ± 3.72a	5.30 ± 3.78a	5.96 ± 3.80a	5.95 ± 1.25a	8.94 ± 5.36a	8.33 ± 3.29a
3.00–3.25 mm	20.45 ± 4.93a	18.90 ± 10.89a	30.08 ± 21.33a	33.84 ± 12.90a	25.43 ± 10.11a	22.30 ± 8.90a
2.75–3.00 mm	27.56 ± 6.78a	35.62 ± 9.02a	31.09 ± 9.56a	34.30 ± 3.49a	30.15 ± 2.53a	26.36 ± 7.78a
2.50–2.75 mm	24.70 ± 3.99a	25.92 ± 7.40a	17.36 ± 9.91a	17.38 ± 9.51a	21.57 ± 6.84a	24.52 ± 8.01a
2.25–2.50 mm	18.99 ± 8.30a	14.26 ± 8.37a	15.52 ± 18.15a	8.53 ± 5.23a	13.91 ± 11.13a	18.48 ± 8.78a
Screenings	8.06 ± 5.46a	3.45 ± 2.18a	6.65 ± 9.33a	2.34 ± 1.13a	5.49 ± 6.53a	5.33 ± 2.89a
**Dimensions**						
Length (mm)	9.09 ± 0.06a	9.08 ± 0.08a	8.77 ± 0.34b	8.80 ± 0.40b	8.96 ± 0.36ab	8.92 ± 0.36ab
Width (mm)	3.60 ± 0.08a	3.61 ± 0.10a	3.60 ± 0.14a	3.74 ± 0.04a	3.68 ± 0.12a	3.66 ± 0.09a
Depth (mm)	2.86 ± 0.06a	2.88 ± 0.14a	2.87 ± 0.15a	2.98 ± 0.07a	2.93 ± 0.16a	2.90 ± 0.10a
2D area (mm^2^)	24.20 ± 0.27a	24.25 ± 0.82a	23.04 ± 0.99a	24.08 ± 1.11a	24.27 ± 0.29a	23.72 ± 0.17a
Circularity	0.56 ± 0.01b	0.57 ± 0.01b	0.59 ± 0.03a	0.59 ± 0.03a	0.59 ± 0.03ab	0.58 ± 0.02ab
**Specific weight and components**						
Specific Weight (kg hL^−1^)	62.73 ± 2.10b	63.76 ± 3.01b	64.12 ± 2.71ab	65.75 ± 1.57ab	66.86 ± 1.91a	66.69 ± 2.52a
Packing Efficiency (%)	52.84 ± 1.95a	51.95 ± 1.93a	51.26 ± 0.09a	52.96 ± 1.21a	52.72 ± 2.85a	53.08 ± 0.80a
Grain Density (g cm^−3^)	1.17 ± 0.02b	1.21 ± 0.02b	1.23 ± 0.05ab	1.25 ± 0.02ab	1.28 ± 0.05a	1.25 ± 0.05a
Grain Volume (cm^−3^)	39.08 ± 0.61a	37.96 ± 2.04a	37.16 ± 0.93a	39.43 ± 0.37a	39.37 ± 2.17a	38.55 ± 0.96a
**Composition**						
Total starch	53.80 ± 0.14a	52.89 ± 1.00a	52.96 ± 2.04a	52.88 ± 0.90a	54.95 ± 2.17a	54.46 ± 2.35a
Amylose (%)	20.17 ± 1.66a	19.16 ± 0.86a	19.24 ± 0.95a	19.40 ± 2.03a	18.68 ± 1.32a	21.05 ± 0.77a
Carbon (%)	39.23 ± 0.89a	39.84 ± 0.02a	40.17 ± 0.85a	39.90 ± 0.12a	39.90 ± 0.04a	39.93 ± 0.15a
Nitrogen (%)	1.67 ± 0.23a	1.84 ± 0.04a	1.81 ± 0.32a	1.90 ± 0.12a	1.61 ± 0.17a	1.76 ± 0.26a
C:N	23.80 ± 2.87a	21.70 ± 0.50b	22.62 ± 3.56a	20.99 ± 1.21b	24.92 ± 2.61a	23.00 ± 3.11b
**Physical-chemical parameters**						
Protein Content (mg/grain)	4.78 ± 0.62b	5.28 ± 0.57a	5.10 ± 0.55b	5.83 ± 0.38a	5.03 ± 0.38b	5.24 ± 0.47a
Starch Content (mg/grain)	24.69 ± 0.26a	24.34 ± 2.66a	24.23 ± 2.78a	25.90 ± 0.18a	27.53 ± 2.73a	26.15 ± 2.49a

^1^ Results which share a letter in a given row are not significantly different from one another after comparison of 95% confidence intervals of the linear mixed model.

**Table 2:** Correlation matrix of Pearson correlation coefficients (r) for grain characteristics.

	Grain weight (mg)	Length (mm)	Width (mm)	Depth (mm)	Volume (mm^3^)	Area (mm^2^)	Perimeter (mm)	Circularity	Grain Density (g cm^−3^)	Packing Efficiency (%)	Specific Weight (kg hL^−1^)	Nitrogen Content (%)	Carbon Content (%)	Starch Content (%)
Grain weight (mg)	1	−0.33	0.89 **	0.84 **	0.83 *	0.32	−0.24	0.64 **	0.48 *	0.62 **	0.84 **	−0.47	−0.18	0.68 **
Length (mm)		1	−0.51 *	−0.53 *	−0.03	0.7 **	0.91 **	−0.8 *	−0.56 *	−0.08	−0.65 **	0.35	0.04	−0.30
Width (mm)			1	0.95 ***	0.79	0.08	−0.46	0.78 ***	0.36	0.57 *	0.76 ***	−0.55 *	−0.38	0.62 **
Depth (mm)				1	0.79	−0.02	−0.53 *	0.8 *	0.27	0.62 **	0.76 ***	−0.49 *	−0.34	0.59 **
Volume (mm^3^)					1	0.48 *	−0.02	0.44	−0.09	0.81 *	0.53 *	−0.43	−0.31	0.63 **
Area (mm^2^)						1	0.80	−0.38	−0.21	0.37	−0.09	0.13	0.02	0.05
Perimeter (mm)							1	−0.86	−0.43	−0.03	−0.55 *	0.42	0.10	−0.30
Circularity								1	0.47	0.34	0.76 ***	−0.53 *	−0.14	0.5 *
Grain Density (g cm^−3^)									1	−0.17	0.67 **	−0.18	0.13	0.23
Packing Efficiency (%)										1	0.53 *	−0.24	−0.20	0.48 *
Specific Weight (kg hL^−1^)											1	−0.41	−0.04	0.64 **
Nitrogen Content (%)												1	0.68 **	−0.76 ***
Carbon Content (%)													1	−0.28
Starch Content (%)														1

“***”, “**”, “*” were significant at *p* < 0.001, *p* < 0.01 and *p* < 0.05 respectively.

## Supplementary Materials

The following are available online at https://www.mdpi.com/2223-7747/9/11/1564/s1, Table S1: Summary of mean values ± standard deviations for plant growth parameters soil moisture across three reps for the three cultivars and two treatment levels used in this study; Table S2: Statistical analyses of the impact of drought and cultivar on grain characteristics using mixed models with rep as a random effect; Table S3: Statistical analyses of the impact of drought and cultivar on grain characteristics using mixed models with rep as a random effect; Table S4: *p*-values associated with the correlation matrix in Table 2.
