# Supplementary material for: Post-Anthesis Water-stressed Barley Maintains Grain Specific Weight Through Altered Grain Composition and Plant Architecture
Source: Plants (Basel). 2020 Nov 13;9(11):1564. doi: 10.3390/plants9111564 (PMC7698198; doi:10.3390/plants9111564)
Supplement: Supplementary file 1 [file plants-09-01564-s001.zip › Table S1.docx]

| **Table S1.** Summary of mean values ± standard deviations for plant growth parameters soil moisture across three reps for the three cultivars and two treatment levels used in this study^a^. | | | | | | |
| --- | --- | --- | --- | --- | --- | --- |
| Plant/Grain Parameters | Octavia | | Concerto | | Sienna | |
|  | Well-watered | Water stress | Well-watered | Water stress | Well-watered | Water stress |
| ***Ear and grain traits*** |  |  |  |  |  |  |
| Grain Weight (mg) | 39.23 ± 0.61a | 39.84 ± 4.32a | 40.17 ± 3.49a | 39.90 ± 0.81a | 39.90 ± 3.42a | 39.93 ± 2.58a |
| Ear Number | 29.67 ± 6.91a | 22.73 ± 8.76b | 31.60 ± 6.05a | 20.47 ± 4.12b | 28.40 ± 8.77a | 22.80 ± 5.35b |
| Ear Length (mm) | 73.42 ± 4.07ab | 75.68 ± 4.48a | 66.57 ± 6.96c | 73.22 ± 7.70bc | 67.51 ± 6.28c | 68.44 ± 4.51bc |
| Grains per pot | 510.67 ± 175.74a | 374.33 ± 152.49b | 534.53 ± 135.16a | 387.07 ± 98.84b | 542.27 ± 152.68a | 412.73 ± 102.50b |
| Grains per ear | 16.99 ± 2.46bc | 16.40 ± 1.55c | 16.83 ± 2.12bc | 18.90 ± 2.67ab | 19.41 ± 2.39a | 18.12 ± 1.69abc |
| Fertility | 0.81 ± 0.03bc | 0.79 ± 0.03c | 0.82 ± 0.03abc | 0.85 ± 0.03ab | 0.86 ± 0.03a | 0.82 ± 0.03abc |
| Days Grain Fill | 49.93 ± 4.06b | 44.40 ± 5.22d | 54.40 ± 4.85a | 46.73 ± 6.15bc | 50.13 ± 3.81ab | 44.40 ± 6.01cd |
| ***Biomass paritioning*** |  |  |  |  |  |  |
| Plant Dry Biomass excluding grains (g) | 24.80 ± 3.96b | 22.26 ± 3.53cd | 24.68 ± 3.71bc | 21.93 ± 2.85d | 29.26 ± 4.56a | 26.47 ± 3.67b |
| Grain Yield (g/pot) | 21.81 ± 9.19a | 17.07 ± 6.99b | 23.51 ± 5.37a | 18.42 ± 4.82b | 24.76 ± 6.60a | 19.27 ± 6.04b |
| Harvest Index | 0.42 ± 0.06ab | 0.39 ± 0.08b | 0.45 ± 0.05a | 0.42 ± 0.05ab | 0.42 ± 0.05ab | 0.38 ± 0.05b |
| ***Soil Moisture*** |  |  |  |  |  |  |
| Volumetric water content (%) | 23.51 ± 0.60a | 13.13 ± 1.18b | 22.99 ± 0.81a | 12.65 ± 0.77b | 23.3 ± 0.78a | 13.04 ± 1.23b |
| Mean theta (mV) | 220.77 ± 7.22a | 114.72 ± 10.02b | 214.61 ± 9.60a | 110.55 ± 6.52b | 218.27 ± 9.43a | 113.93 ± 10.54b |
| ^a^ Results which share a letter in a given row are not significantly different from one another after comparison of 95% confidence intervals of the linear mixed model. | | | | | | |
