# Supplementary material for: Post-Anthesis Water-stressed Barley Maintains Grain Specific Weight Through Altered Grain Composition and Plant Architecture
Source: Plants (Basel). 2020 Nov 13;9(11):1564. doi: 10.3390/plants9111564 (PMC7698198; doi:10.3390/plants9111564)
Supplement: Supplementary file 1 [file plants-09-01564-s001.zip › Table S2.docx]

| Table S2. Statistical analyses of the impact of drought and cultivar on grain characteristics using mixed models with rep as a random effect. | | | |
| --- | --- | --- | --- |
| Response Variable | Treatment*Cultivar | Cultivar | Treatment |
| *Ear and grain traits* |  |  |  |
| Grain weight (mg) | ns | ns | ns |
| Ear Number | ns | ns | 8.20E-10 |
| Ear Length (mm) | ns | 6.67E-05 | 0.00699 |
| Grains per pot | ns | ns | 1.66E-08 |
| Grains per ear | 0.01653 | * | * |
|  |  |  |  |
| *Biomass partitioning* | |  |  |
| Plant Dry Biomass (g) | ns | 8.89E-09 | 3.41E-05 |
| Grain Yield (g/pot) | ns | ns | 0.000101 |
| Harvest Index | ns | 0.04617 | 4.11E-03 |
|  |  |  |  |
| *Plant Growth* |  |  |  |
| Mean theta (mV) | ns | ns | 2.20E-16 |
| Fertility | 0.03789 | * | * |
| Days Grain Fill | ns | 0.00534 | 1.59E-09 |
