# Supplementary material for: Post-Anthesis Water-stressed Barley Maintains Grain Specific Weight Through Altered Grain Composition and Plant Architecture
Source: Plants (Basel). 2020 Nov 13;9(11):1564. doi: 10.3390/plants9111564 (PMC7698198; doi:10.3390/plants9111564)
Supplement: Supplementary file 1 [file plants-09-01564-s001.zip › Table S3.docx]

| Table S3. Statistical analyses of the impact of drought and cultivar on grain characteristics using mixed models with rep as a random effect. | | | |
| --- | --- | --- | --- |
| Response Variable | Treatment*Cultivar | Cultivar | Treatment |
| *Size Classes* |  |  |  |
| >3.25 mm | ns | ns | ns |
| 3.00-3.25 mm | ns | ns | ns |
| 2.75-3.00 mm | ns | ns | ns |
| 2.50-2.75 mm | ns | ns | ns |
| 2.25-2.50 mm | ns | ns | ns |
| Screenings (%) | ns | ns | ns |
| *Dimensions* |  |  |  |
| Length (mm) | ns | 0.04304 | ns |
| Width (mm) | ns | ns | ns |
| Depth (mm) | ns | ns | ns |
| 2D area (mm^2^) | ns | ns | ns |
| Circularity | ns | 0.0087 | ns |
| *Specific weight and components* | |  |  |
| Specific Weight (kg hl^-1^) | ns | 0.003 | ns |
| Packing Efficiency (%) | ns | ns | ns |
| Density (g cm^-3^) | ns | 0.00857 | ns |
| *Composition* |  |  |  |
| Total starch | ns | 0.02794 | ns |
| Amylose (%) | 0.03979 |  |  |
| Carbon (%) | ns | ns | ns |
| Nitrogen (%) | ns | ns | 0.04456 |
| C:N | ns | ns | 0.02958 |
| *Physical-chemical parameters* |  |  |  |
| Protein Content (mg/grain) | ns | ns | 0.01215 |
| Starch Content (mg/grain) | ns | ns | ns |
