# Supplementary material for: Post-Anthesis Water-stressed Barley Maintains Grain Specific Weight Through Altered Grain Composition and Plant Architecture
Source: Plants (Basel). 2020 Nov 13;9(11):1564. doi: 10.3390/plants9111564 (PMC7698198; doi:10.3390/plants9111564)
Supplement: Supplementary file 1 [file plants-09-01564-s001.zip › Table S4.docx]

|  |  |  |  |  |  |  |  |  |  |  |  |  |  |  |
| --- | --- | --- | --- | --- | --- | --- | --- | --- | --- | --- | --- | --- | --- | --- |
|  | Grain weight  (mg) | Length  (mm) | Width  (mm) | Depth  (mm) | Volume  (mm^3^) | Area  (mm^2^) | Perimeter  (mm) | Circularity | Density  (g cm^-3^) | Packing  Efficiency (%) | Specific Weight  (kg hl^-1^) | Nitrogen  Content (%) | Carbon  Content (%) | Starch  Content (%) |
| Grain weight (mg) | 0 | 0.177 | 0.00533 | 0.00916 | 0.0125 | 0.197 | 0.328 | 0.00404 | 0.0447 | 0.00615 | 0.00885 | 0.0509 | 0.465 | 0.00199 |
| Length (mm) |  | 0 | 0.0321 | 0.0222 | 0.903 | 0.00116 | 0.00141 | 0.0482 | 0.0152 | 0.76 | 0.00369 | 0.159 | 0.886 | 0.234 |
| Width (mm) |  |  | 0 | 0.000185 | 0.0592 | 0.756 | 0.0538 | 0.00013 | 0.14 | 0.0144 | 0.00024 | 0.019 | 0.123 | 0.00573 |
| Depth (mm) |  |  |  | 0 | 0.063 | 0.937 | 0.0232 | 0.0468 | 0.28 | 0.00644 | 0.000237 | 0.0368 | 0.162 | 0.00989 |
| Volume (mm^3^) |  |  |  |  | 0 | 0.0425 | 0.942 | 0.0689 | 0.729 | 0.0258 | 0.0225 | 0.0758 | 0.215 | 0.00472 |
| Area (mm^2^) |  |  |  |  |  | 0 | 0.0518 | 0.125 | 0.4068 | 0.135 | 0.722 | 0.609 | 0.933 | 0.855 |
| Perimeter (mm) |  |  |  |  |  |  | 0 | 0.0127 | 0.0765 | 0.908 | 0.0169 | 0.0853 | 0.68 | 0.219 |
| Circularity |  |  |  |  |  |  |  | 0 | 0.0473 | 0.172 | 0.000281 | 0.025 | 0.579 | 0.0342 |
| Density (g cm^-3^) |  |  |  |  |  |  |  |  | 0 | 0.512 | 0.002445 | 0.4654 | 0.601 | 0.361 |
| Packing Efficiency (%) |  |  |  |  |  |  |  |  |  | 0 | 0.0224 | 0.341 | 0.429 | 0.0445 |
| Specific Weight (kg hl^-1^) |  |  |  |  |  |  |  |  |  |  | 0 | 0.0931 | 0.887 | 0.00419 |
| Nitrogen Content (%) |  |  |  |  |  |  |  |  |  |  |  | 0 | 0.00197 | 0.00025 |
| Carbon Content (%) |  |  |  |  |  |  |  |  |  |  |  |  | 0 | 0.26 |
| Starch Content (%) |  |  |  |  |  |  |  |  |  |  |  |  |  | 0 |

Table S4. p-values associated with the correlation matrix in Table 2.
